# Supplementary material for: Layer- and cell type-selective co-transmission by a basal forebrain cholinergic projection to the olfactory bulb
Source: Nat Commun. 2017 Sep 21;8:652. doi: 10.1038/s41467-017-00765-4 (PMC5608700; doi:10.1038/s41467-017-00765-4)
Supplement: Supplementary file 1 — Supplementary Information [file 41467_2017_765_MOESM1_ESM.pdf]

File Name: Supplementary Information
Description: Supplementary Figures

File Name: Peer Review File

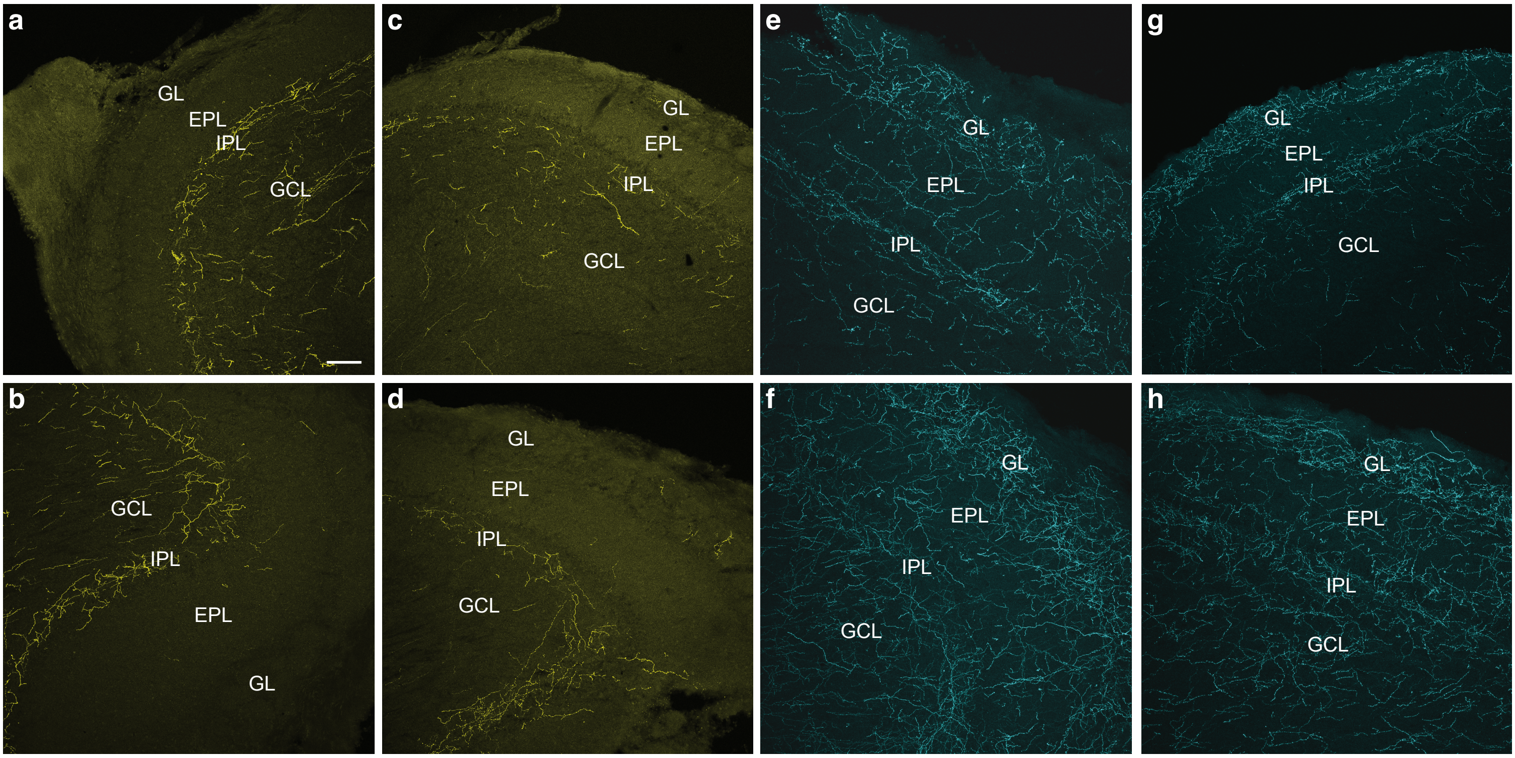

Supplementary Figure 1. Preferential innervation of the IPL by VGLUT3^{+} neurons in the HDB. (**a-d**) MOB innervation by VGLUT3^{+} neurons in the HDB, labeled by Cre-dependent expression of ChR2-EYFP following virus injection into the HDB of VGLUT3^{Cre} mice. (**e-h**) MOB innervation by cholinergic neurons in the HDB, labeled by Cre-dependent expression of ChR2-EYFP following virus injection into the HDB of ChAT^{Cre} mice. Sections from eight total representative mice are shown. See **Fig. 2** for additional examples.

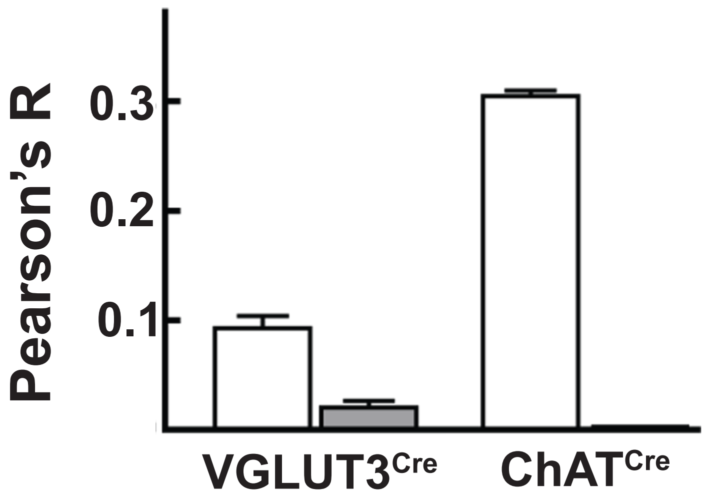

Supplementary Figure 2. The Pearson's Correlation Coefficient (R) for VAChT and the ChR2-EYFP in IPL of VGLUT3^{Cre} mice injected with AAV9 EF1a-DIO-ChR2-EYFP in the HDB is low (R = 0.09 +/- 0.01, open bar), but is still significantly larger than the same correlation with one channel in the image flipped (R = 0.02 +/- 0.01; p = 0.006; n = 4, gray bar). However, the Pearson's R value for VAChT and the ChR2-EYFP in IPL fibers of VGLUT3^{Cre} mice injected with AAV9 EF1a-DIO-ChR2-EYFP in the HDB is also not exceedingly high (R = 0.31 +/- 0.01, n = 2, open bar), suggesting that the two fluorescent molecules do not overlap well in fiber terminals. Additionally, the correlation coefficient calculated for the fibers in ChAT^{Cre} mice is approximately 3x higher than in VGLUT3^{Cre} mice, suggesting that approximately one-third of the cholinergic fibers that course through the IPL are derived from the VGLUT3^{Cre} population. Together with our other data, these findings indicate that the VGLUT3^{Cre} cholinergic fibers make up a subset of cholinergic fibers even within the IPL.

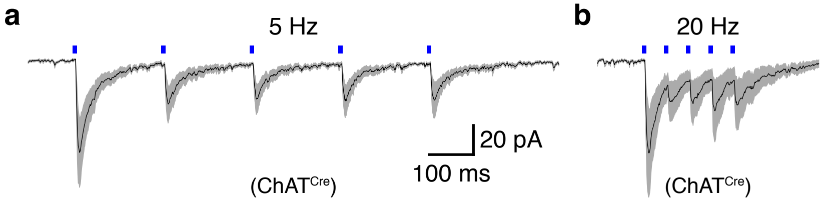

Supplementary Figure 3. Short-term plasticity of BF input to GL-dSACs. (**a**,**b**) Repetitive photostimulation (blue lines) of HDB projections at 5 Hz (**a**, n=6) and 20 Hz (**b**, n=5) in acute slices from ChAT^{Cre} mice evoked EPSCs in GL-dSACs with high probability and moderate short-term depression.
